# Molecularly Imprinted Polymer-Based Luminescent Chemosensors

**DOI:** 10.3390/bios13020295

**Published:** 2023-02-19

**Authors:** Ruoyang Liu, Chi-Chiu Ko

**Affiliations:** Department of Chemistry and State Key Laboratory of Marine Pollution, City University of Hong Kong, Tat Chee Avenue, Kowloon, Hong Kong 518057, China

**Keywords:** luminescent, molecularly imprinted polymer, chemosensor, transition metal complex, quantum dots, organic dye

## Abstract

Molecularly imprinted polymer (MIP)-based luminescent chemosensors combine the advantages of the highly specific molecular recognition of the imprinting sites and the high sensitivity with the luminescence detection. These advantages have drawn great attention during the past two decades. Luminescent molecularly imprinted polymers (luminescent MIPs) towards different targeted analytes are constructed with different strategies, such as the incorporation of luminescent functional monomers, physical entrapment, covalent attachment of luminescent signaling elements on the MIPs, and surface-imprinting polymerization on the luminescent nanomaterials. In this review, we will discuss the design strategies and sensing approaches of luminescent MIP-based chemosensors, as well as their selected applications in biosensing, bioimaging, food safety, and clinical diagnosis. The limitations and prospects for the future development of MIP-based luminescent chemosensors will also be discussed.

## 1. Introduction

Chemosensors are composed of a receptor functional moiety and a reporter, which can produce a detectable signal response, such as absorption, luminescent or electrical, to reflect the binding of an analyte with the receptor via non-covalent host-guest interactions. For an ideal chemosensor, the host-guest interaction must be highly specific to the targeted analyte and preferably with high binding affinity to achieve high selectivity and sensitivity [1,2,3,4]. As a result, receptor design is crucial for the successful development of chemosensors but is usually the most challenging task. On the other hand, the high sensitivity and non-destructive nature of luminescence, both fluorescence and phosphorescence, are attractive features for a chemosensor’s reporter. In this context, the development of luminescent molecular devices, such as switches, sensors, and molecular machines, has been an active area of research in supramolecular photochemistry. To design luminescent chemosensors, a luminophore is connected to the receptor so that the binding event between the receptor (host) and the targeted analyte (guest) induce a change of its emission properties, which serve as the read-out signals for the qualitative and quantitative determination of the analyte [4,5,6,7,8].

The extensive developments of molecularly imprinted polymers (MIPs) have provided an effective way for preparing materials with receptor sites, which are highly specific to the molecular template used in the preparation of the MIPs or molecules with high structural similarity, including the distributions of the functional group moieties, hydrophobicity, and polarity. In addition to the highly selective binding receptors, MIPs are also well-known for their inherent mechanical and chemical stability, strong binding affinity, short development time, and low cost with well-developed preparation strategies [9,10,11]. Generally, the in-situ polymerization of the monomer, functional monomer, and crosslinker in the presence of a target template molecule is employed to synthesize MIPs. The functional monomers are designed with substituents or groups to interact with template molecules. Due to the interactions, such as hydrogen bonds or non-covalent interactions, between the functional monomer and the template, the reaction mixtures are pre-organized such that the template molecules are surrounded by functional monomers. Upon cross-linking polymerization, the template molecules are encapsulated in the polymer matrices. After the removal of the template molecules, template-shaped cavities with complementary functional groups, sizes, and shapes best-fitting the template molecules become the highly selective receptor site for the template molecules. These molecular imprinting technologies have been well-established to develop materials with highly selective receptors and thus widely exploited in many significant applications in different fields, including biosensors, solid-phase extraction, chromatographic separation, catalysis, drug-controlled release, chemical analysis, and hybrid with organic polymers (such as MOFs) to make composite materials [12,13,14,15,16]. The merits of the imprinting effect of MIP are characterized by the imprinting factor, binding capacity, and selectivity. Amongst, the imprinting factor is determined by comparing the amount of bound analyte by MIP and its corresponding nonimprinted polymer (NIP), the selectivity is determined by the outcome of rebinding assay compared between the target analyte and structural analog, and the equilibrium binding capacity is normally measured by HPLC, GS-MS, and UV-vis. The details of measurement have been described in a recent review [17].

The use of MIPs in the development of chemosensors offers another advantage, as the polymeric systems are well-known to provide collective responses to enhance their sensitivity by “amplifying” the signals compared to single molecular systems [18]. The amplifying effect enables the detection of the binding event by gravimetric methods using a highly sensitive quartz microbalance [19]. However, the use of an expensive and sophisticated microbalance has limited their applications in the research laboratory. To extend their applications as portable or real-time monitoring sensors for on-site utilization, a signal-transducing component has been introduced to the receptor-containing polymer. Optical signaling, including absorption and luminescence, is one of the preferable means as it is easily detected with portable devices or even the naked eye. For example, chemosensors for aromatic explosives such as 2,4,6-trinitrotoluene (TNT) and 2,4-dinitrotoluene (DNT) can be developed by conjugation of a suitable fluorophore into a polymer main chain [20]. Polymer-based luminescent chemosensors for metal cations such as lead(II), palladium(II), and iron(II) ions have also been developed [21,22,23]. In these chemosensors, the binding of the target molecules/ions onto the polymer can be reflected by the quenching of fluorescence of the fluorophore. Apart from “turn-off” fluorescent chemosensors, the more sensitive “turn-on” polymer-based chemosensors have also been developed [24,25].

**Figure 1:**
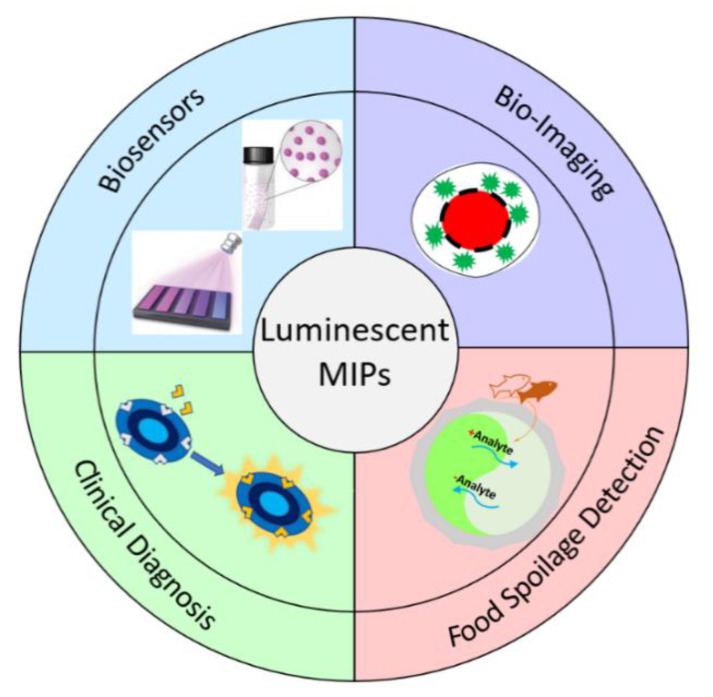
Schematic illustration of potential applications of luminescent MIPs.

Given the ease of developing highly selective receptors from MIPs, the amplification effect in polymer-based sensors as well as the highly sensitive luminescent detection, the recent advances in luminescent MIP-based sensors and devices are reviewed in this article. The design strategies and classifications of luminescent MIP-based chemosensors and their selected applications in biosensing, bioimaging, food safety, and clinical diagnosis (Figure 1) will also be discussed. 

## 2. Molecular Imprinting Strategy

As mentioned above, MIPs, as selective sorbents, are prepared from a mixture containing at least two essential components, namely functional monomers for interacting with the template or structurally-related molecule that acts as a template. The design concept of MIP was first reported and demonstrated using silica matrices by Polyakov’s seminal work ninety years ago. Different strategies for preparing MIP have been rapidly developed over the past few decades due to the exponential growth and development of organic polymers [26].

**Figure 2:**
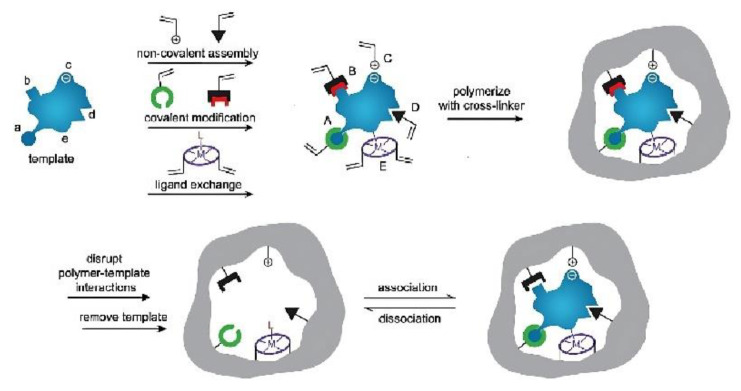
Schematic representation of the molecular imprinting polymerization process. Adapted with permission [26]. Copyright 2006, John Wiley & Sons, Ltd.

In the syntheses of MIPs, the template-monomer(s) adduct was formed in the reaction mixture via one or more intermolecular interactions, such as reversible covalent bond formation, semi-covalent or non-covalent interactions (electrostatic affinity, van der Waals interactions, hydrophobic forces, and coordination with a metal center). The subsequent polymerization is performed in the presence of an initiator and crosslinker in the solvent. With crosslinking polymerization, the orientation of monomers and the functional sites that bound with the template molecule are fixed and rigidified by crosslinking units in a three-dimensional network to avoid their random movement. Subsequent removal of the template molecules from the MIP matrix leaves robust binding sites that are highly selective to the template molecule and structurally similar molecules having the same functional groups (Figure 2). These highly selective receptors are usually referred to as the “imprinted” sites of MIP [27].

In general, MIPs can be prepared by copolymerization reactions of a combination of the most commonly used building blocks (monomer and crosslinker) in the presence of template molecules, initiators, and solvents, as summarized in Table 1. Although different types of MIPs are designed based on the same principle as illustrated in Figure 2, their imprinting performance is strongly dependent on the building blocks, types of target molecules as well as the polymerization conditions, including temperature, initiator, and solvents [28,29]. The molecular design of the monomer, crosslinker, template, and polymerization conditions to enhance the imprinting factors have been extensively reviewed [28,29,30,31,32,33,34]. In these reviews, the development of functional monomers with complementary functional moieties to form donor-acceptor interactions with the template molecules [30,31,32] and the effects of crosslinkers and solvents on controlling the recognition site as well as polymer morphology are discussed in detail [33,34].

With the highly selective binding sites, MIPs have found a wide range of applications such as solid-phase extraction (SPE), sensors, membranes, catalysis, synthesis, and drug delivery [35,36]. Moreover, the high thermal stability and structural rigidity of MIPs enable their use under harsh conditions. Owing to the unique features of structure predictability and recognition specificity, molecularly imprinted polymers are universally applied in sample pretreatment, chromatographic separation, and chemical/biological sensing [37,38]. With the recent advance in surface imprinting technology together with the hollow porous polymer synthesis, MIPs with high adsorption capacity and high imprinting efficiency, good morphology, uniform size, and ideal surface properties can be obtained [39]. The surface MIPs on hollow porous polymers are ideal to be used as sorbents and stationary phases for sample pretreatment and chromatography [40]. Further enhancements of the surface areas, interfacial properties, and binding capacity have also been achieved by incorporating the composite imprinting strategy into sol-gel processes for nanomaterials and nanoimprinting [41,42]. 

## 3. Design Strategies of Luminescent MIPs for Chemosensing Applications

Luminescence detection has been providing a significant and attractive approach for numerous chemical, biological, and environmental species because of its high sensitivity, non-destructive nature, and stability [4,5,6,7,8]. As demonstrated in recent decades, many luminescent MIP-based sensors possess high sensitivity of luminescence detection and high selectivity of MIP recognition [43,44,45,46]. 

For emissive analytes, their MIP-based chemosensors can be non-emissive and thus are generally prepared using the standard method for MIPs using the analyte as the template. As the luminescent properties of the analytes would change upon binding with the imprinted sites of the MIP due to the changes in the micro-environment and the electronic properties resulting from the binding interactions, such changes can be used for the qualitative and quantitative determination of the analyte. Selected examples include MIP-based sensors for enrofloxacin and fluoroquinolone analogs [47] and rhodamine derivatives [48]. As most of the target analytes are non-emissive, there are only a few reports on this type of MIP-based luminescent chemosensors.

For non-emissive analytes, luminescent chemosensing can be achieved by displacement or competitive assay using a non-emissive MIP with a luminescent-labeled analyte as the template [49,50]. However, this design cannot be widely applied as luminescent labeling of the target analyte can be extremely challenging or even impossible. The introduction of luminophore into the MIP to report the binding event of the imprinted site represents a more versatile design strategy for developing luminescent MIP-based chemosensors [51]. With this strategy, different types of luminophores, including fluorescent organic compounds, luminescent transition metal complexes, nanoparticles, and quantum dots with different emission characteristics, can be rationally chosen to successfully develop the luminescent MIP-based chemosensors [43,44,45,46,52,53]. 

Early design of luminescent MIPs is mainly based on the physical entrapment or chemical modification of the MIPs through the addition of luminescent dye or polymerizable organic fluorescent-dye-containing monomers in the preparation of MIPs, respectively [54,55]. However, most of the fluorescent moieties in the MIPs do not show any emission responses to the binding event of the imprinted receptors because they are randomly embedded in MIP, and most of them do not have any electronic communication with the receptor moieties. To increase the luminescent responses to the binding event of the imprinted receptors, monomers with both fluorescent and binding moieties have been used [56]. However, the syntheses of the highly functionalized monomer are usually complicated, and the fluorescent responses of the MIPs are still not as strong as the receptor-containing fluorescent monomer in the solution state. To further enhance the emission changes of the MIPs upon binding with the target guest molecules at the receptors, a new design of luminescent MIPs by chemical modification of the imprinted receptor sites of the non-emissive MIPs with the emissive dyes has been reported [57]. With the recent development of luminescent nano-particles and nano-clusters, which show intense emission with narrow emission band, large Stokes shift, and readily tunable emission characteristics [58], new designs of luminescent MIPs with core-shell structures have been prepared by surface imprinting polymerization on these luminescent nano-particles. However, the successful signal transduction of the binding event of the surface-imprinted polymer to perturb the emission of the nano-particles remains challenging. 

### 3.1. Using Luminescent Monomer as a Building Block of MIPs 

**Figure 3:**
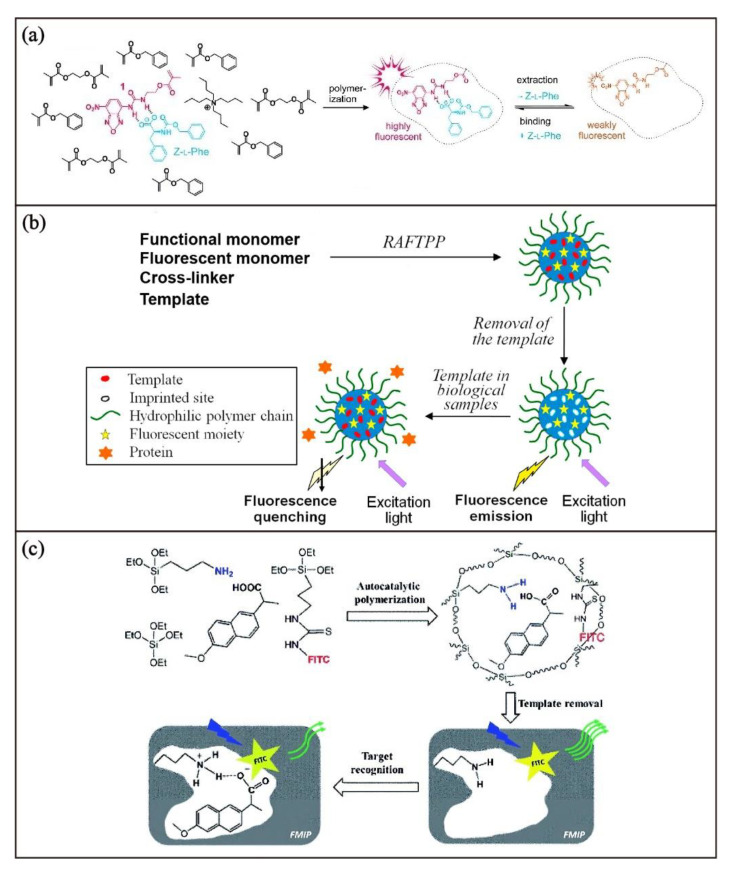
Schematic illustration of the synthesis of (**a**) fluorescent MIP for N-carbobenzyloxy-l-phenylalanine detection. Adapted with permission [56]. Copyright 2013, Wiley-VCH. (**b**) Hydrophilic fluorescent tetracycline-imprinted MIP nanoparticles for drug sensing. Adapted with permission [59]. Copyright 2015, Elsevier. (**c**) Fluorescent MIP for naproxen sensing. Adapted with permission [60]. Copyright 2021, Royal Society of Chemistry.

Through the copolymerization of luminescent monomer in the preparation of MIPs, emissive MIPs with the emission properties derived from the luminophore of the monomer can be obtained. This method has been extensively explored in the past decade for the development of luminescent chemosensors. For example, Rurack and coworkers [56] designed a fluorescent monomer containing both urea-receptor and nitrobenzoxadiazole fluorophore to prepare a fluorescent MIP. Based on the binding of the urea group with the carboxylate, fluorescent MIP with imprinting sites specific for N-carbobenzyloxy-l-phenylalanine (Z-L-Phe) can be prepared by reversible addition-fragmentation chain-transfer (RAFT) polymerization of the fluorescent monomer, ethylene glycol dimethacrylate (EDGA) and benzyl methacrylate (Figure 3a). With the hydrogen-bonding interactions between Z-L-Phe and the urea functional group in the binding site, a strongly bonded complex adduct is formed to avoid the formation of the non-emissive deprotonated species. As a result, the presence of Z-L-Phe leads to a pronounced enhancement of fluorescence, which can be used for qualitative and quantitative analysis of Z-L-Phe. It is worth noting that the solvent used for the RAFT polymerization also plays an important role in the binding affinity and sensing responses of the resulting MIP.

Another example is illustrated in fluorescent tetracycline-imprinted polymers reported by Zhang and coworkers [59]. By copolymerization of a fluorescent monomer (2-hydroxyethyl anthrancene-9-carboxylate) methacrylate (AnHEMA), methacrylic acid monomer, and EDGA crosslinker in the presence of tetracycline as a template, fluorescent MIP for tetracycline (Tc-MIP) can be obtained. However, the tetracycline-binding of such MIP is limited in organic solvents due to its hydrophobicity, and thus the fluorescent response for the chemosensory application cannot function in aqueous and biological media. To introduce the hydrophilicity of the fluorescent Tc-MIP so that it can function in an aqueous medium and undiluted bovine serum, poly(2-hydroxyethyl methacrylate) (PHEMA) as hydrophilic polymer brushes grafted on the surface of fluorescent Tc-MIP nanoparticles were prepared. The hydrophilic polymer brushes were introduced by the addition of a well-defined PHEMA with a dithioester end group in the RAFT precipitation copolymerization reaction (Figure 3b). With the PHEMA-grafted fluorescent Tc-MIP, significant fluorescence quenching resulting from the binding of tetracycline could be observed in the biological milieu. Apart from organic fluorescent MIPs, silica-based fluorescent MIPs designed using a similar synthetic strategy with a fluorescent monomer have also been reported [60]. By one-pot copolymerization of a fluorescent monomer containing fluorescein fluorophore (FITC) and amino-receptor containing monomer, 3-aminopropyltriethoxysilane, and the tetraethoxysilane in the presence of naproxen as the template under a catalyst-free condition, fluorescent silica-based MIP nanoparticles showing fast and specific sensing luminescent response towards naproxen can be obtained (Figure 3c).

Although wide varieties of organic fluorescent functional monomers are observed to have good compatibility with polymeric materials and relatively high fluorescent intensities, their universal applications are hindered by the broad and tailing emission peaks. These limitations are more pronounced in MIPs with fluorophores and receptors derived from two separated monomers, in which the emission of some of the fluorophores is unaffected by the binding event. Moreover, poor photostability and photobleaching of these MIPs have also been reported [46].

### 3.2. Chemical Surface Functionalization with a Luminophore

MIPs can be made luminescent through immobilization strategies by attaching luminescent signaling elements. Chemical surface functionalization methods are applied to provide covalent binding sites for exterior luminescent moieties. The commonly used functional groups for covalent immobilization include thiol, amino, carboxyl, hydroxyl, vinyl, and azide groups [61,62,63]. With these functional groups on the MIPs, luminophores can be covalently immobilized on the surface, including the molecular recognition cavities, or directed to the designed sites through click reactions and post-imprinting modification. This strategy has been extensively explored since 2010 [57]. Upon grafting luminescent labels, the luminescent signals can be detected by emission spectroscopy based on the intrinsic properties of luminescent signaling elements [57,64,65].

**Figure 4:**
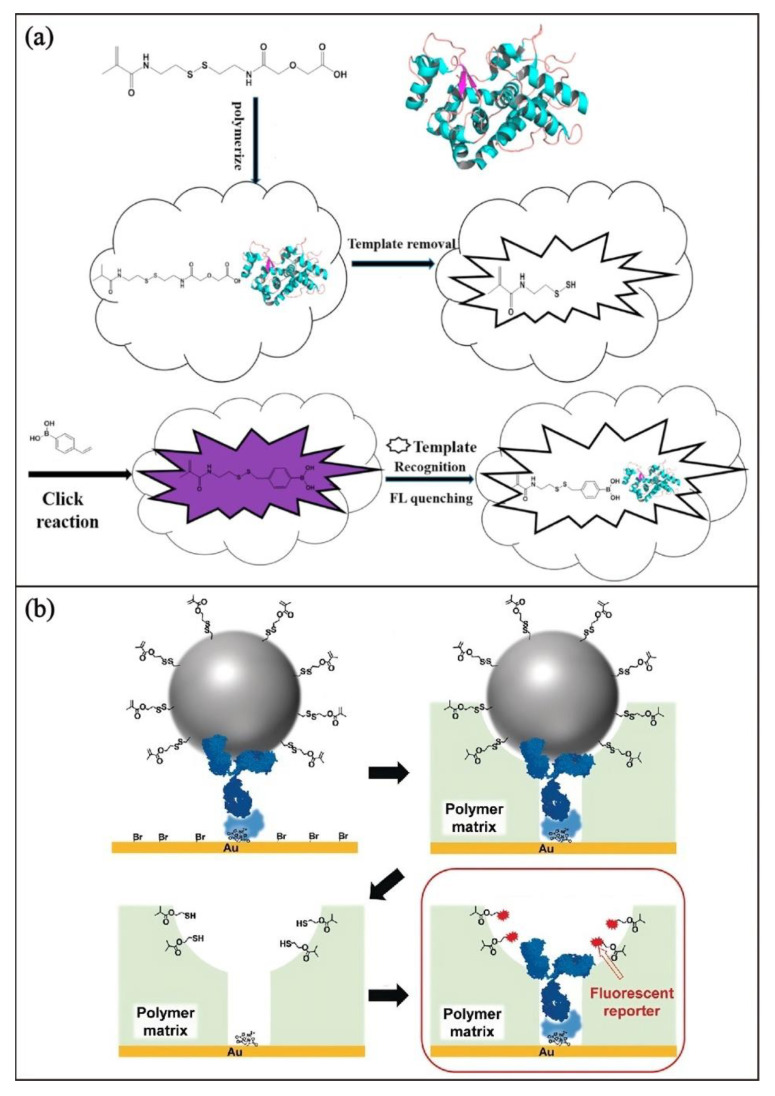
Schematic illustration of the preparation of (**a**) luminescent protein imprinted polymer for glycoprotein detection. Adapted with permission [66]. Copyright 2017, Elsevier. (**b**) Fluorescent sensing platform for exosome detection. Adapted with permission [67]. Copyright 2019, Wiley-VCH.

Wang and coworkers [66] developed a fluorescent protein-imprinted polymer sensor for the fast detection of glycoproteins. In this study, the thiol groups are used for linkage with 4-vinyl phenylboronic acid through click reaction to serve as recognition and luminescent signaling moiety. The 4-vinyl phenylboronic acid forms the imprinted recognition cavity as well as extends π-conjugation to enhance the luminescence properties (Figure 4a). Takeuchi and coworkers [67] reported a fluorescent sensing platform for exosome detection. Using antibody-conjugated exosomes with polymerizable methyacryloyl group as templates, MIPs with imprinted cavities can be prepared. Subsequent removal of the exosomes would leave the imprinted sites with thiol groups. Fluorophores can then be directed to the thiol groups on the cavity to form an immobilization linkage and to serve as a reporter for the binding event between the cavity and the exosomes (Figure 4b).

With luminophores attached through covalent bonding, a chemical surface functionalization is a promising tool for translating recognition events into luminescent signals. However, maintaining the integrity of the binding cavities after chemical surface functionalization so as not to weaken the selectivity remains a challenging issue.

### 3.3. Physical Entrapment

Since the first report of luminescent lanthanide-based copolymer sensors by Murray and coworkers [54,68], lanthanide metal ions/complexes, especially those of terbium(III) and europium(III), have been extensively used as luminophores for developing luminescent MIP-based sensors. The popularity of lanthanides can be attributed to their unique luminescent properties, such as narrow emission bandwidths and extremely long emission lifetimes. Lanthanide ions can be incorporated into the polymer matrix through physical entrapment. After physical entrapment, the lanthanide ions are surrounded by the rigid polymeric matrix, and thus they are highly stable. Moreover, they also exhibit similar photophysical properties as in the solution state [69].

**Figure 5:**
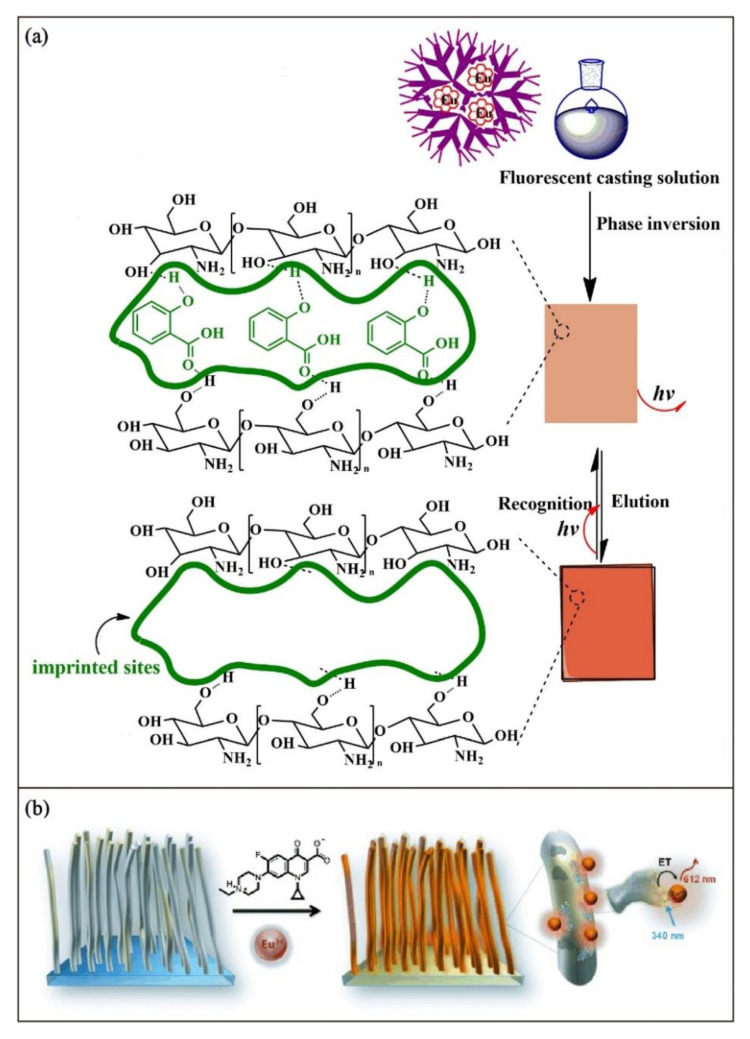
Schematic illustration of (**a**) preparation and sensing mechanism of fluorescence molecularly imprinted membrane for salicylic acid detection. Adapted with permission [70]. Copyright 2019, Elsevier. (**b**) Fabrication of enrofloxacin-imprinted nanofilament entrapped with Eu(III) ions for enrofloxacin detection. Adapted with permission [71]. Copyright 2013, Wiley-VCH.

For example, Pan and coworkers [70] incorporated [Eu(TTA)_3_phen] into poly(amidoamine) dendrimer to serve as a luminescent additive dispersing on the surface of the molecularly imprinted membrane for the selective recognition of salicylic acid (Figure 5a). Moreno-Bondi and coworkers [71] developed a molecularly imprinted nanofilament polymer with physically entrapped Eu(III) ions for fluorescent sensing of enrofloxacin. In their report, the entrapped Eu(III) ions can be derivatized by enrofloxacin in the solution state to form a europium-enrofloxacin complex. By the detection of the change in the emission intensity, in-situ monitoring of the enrofloxacin can be achieved (Figure 5b). As the luminescent lifetimes of lanthanides are significantly longer than the polymer backbone, time-resolved emission spectroscopy can be used to discriminate the background emission from the polymer backbone.

### 3.4. Encapsulation

**Figure 6:**
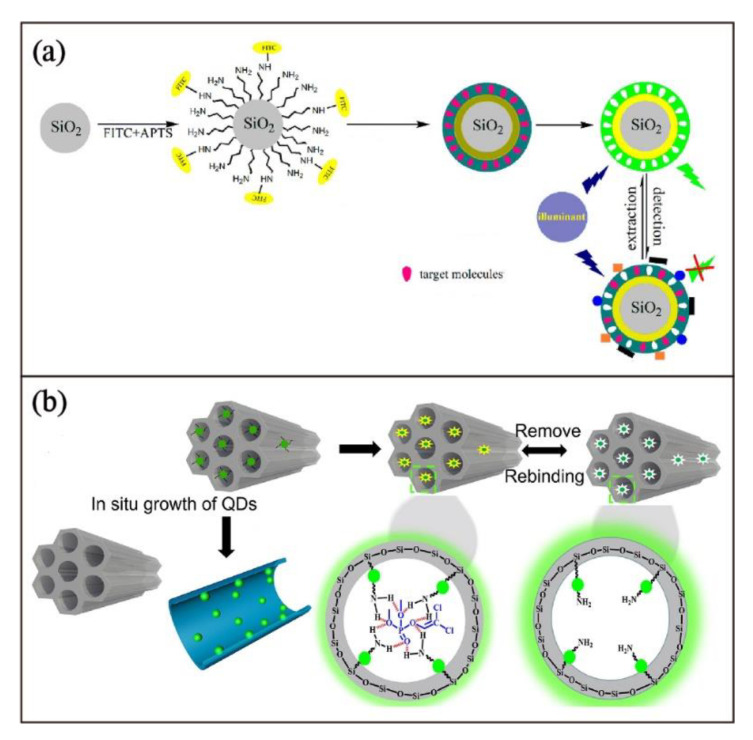
Schematic illustration of (**a**) preparation of fluorescent core-shell MIP sensor for λ-cyhalothrin. Adapted with permission [72]. Copyright 2015, American Chemical Society. (**b**) Fabrication of perovskite QDs in the mesopores of imprinted silica polymer for 2,2-dichlorovinyl dimethyl phosphate detection. Adapted with permission [75]. Copyright 2020, Elsevier.

Luminescent MIPs can also be fabricated by encapsulation with luminescent micro- or nano-particles, which are produced by emissive nanomaterials or immobilization of luminophores on the solid-supported micro- or nano-particles. These types of emissive MIPs are commonly prepared by surface molecularly imprinting techniques on solid luminescent substrates. For example, Yan and coworkers [72] fabricated a fluorescent core-shell MIP sensor for selective detection of λ-cyhalothrin. The imprinting sites are formed on the surface of the modified SiO_2_ beads with a fluorescent dye FITC as the fluorescent reporter. To prepare SiO_2_ spheres with FITC, FITC is first conjugated with 3-aminopropyltriethoxysilane and then coated on SiO_2_ spheres. The resulting core-shell fluorescent MIP sensor can quantify λ-cyhalothrin with a wide range of 10–60 nM and a detection limit of 9.17 nM, according to the Stern–Volmer quenching study (Figure 6a).

For emissive nanomaterials, different strategies were used to incorporate these materials in the preparation of luminescent MIPs. These include the addition of nanoparticle emitters in the conventional preparation of MIPs to give nanocomposite through chemical bonding or physical effects [73]; surface imprinting polymerization as a coating layer with imprinted cavities on the surface of emissive nanomaterials similar to the example described in Figure 6a [74] or on the surface of encapsulated luminescent inorganic nanomaterials (Figure 6b) [75]. The last strategy is exemplified in a recently reported molecularly imprinted silica polymer on the perovskite quantum dots (QDs) encapsulated mesoporous silica [74]. In this study, the emission properties of the QDs can be used for the detection of 2,2-dichlorovinyl dimethyl phosphate. 

## 4. Classifications of Luminescent MIPs Based on the Nature of Luminophore

### 4.1. Luminescent Transition Metal Complexes-Functionalized MIPs

**Figure 7:**
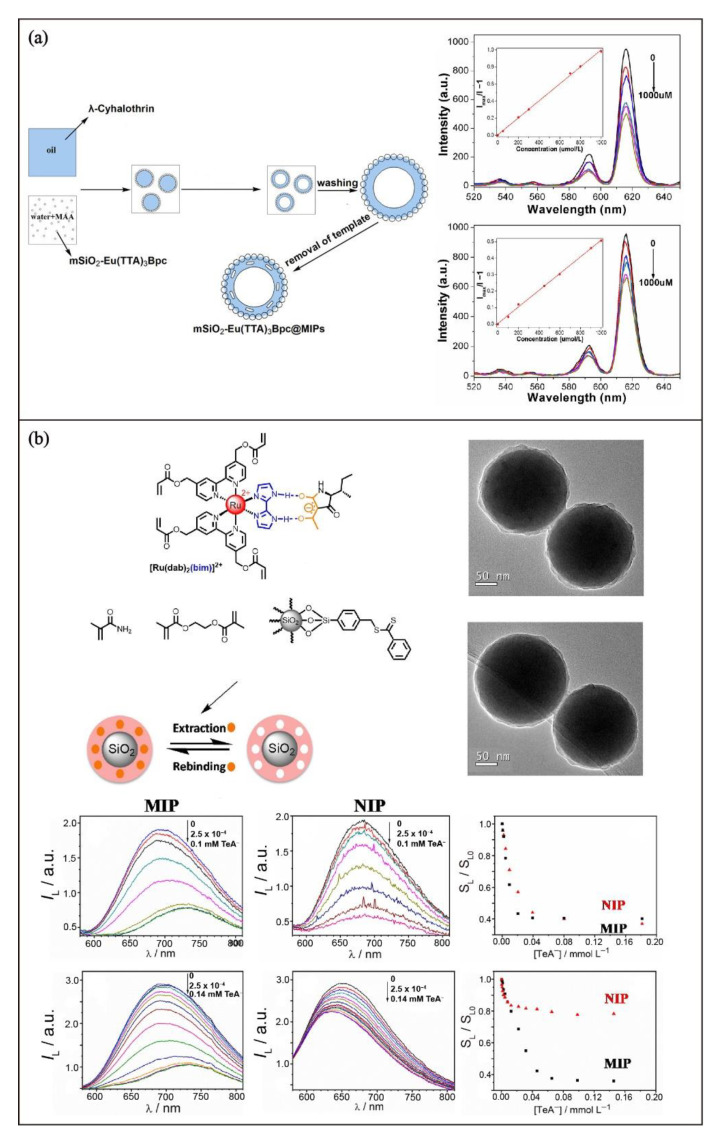
(**a**) Preparation of the silica-MIP modified with Eu(III) complexes and the emission quenching spectra of the MIP and non-imprinted polymer (NIP) for λ-cyhalothrin sensing. Adapted with permission [83]. Copyright 2013, American Chemical Society. (**b**) Fabrication of luminescent core-shell silica nanoparticle-MIP functionalized with Ru(II) complexes and the emission spectral changes in the tenuazonic acid sensing study. Adapted with permission [79]. Copyright 2021, Elsevier.

Different immobilization strategies, including the use of transition metal complexes with alkene monomer-containing ligands [76,77,78,79], dithiobenzoate substituted ligands for RAFT polymerization [80], physically entrapping into polymer network [81], and post-functionalization by surface chemical modification [61,62,63,64,65,66,67], have been used to prepare MIPs functionalized with luminescent transition metal complexes. Amongst different types of luminescent transition metal complexes, luminescent lanthanide complexes, especially those of Eu(III) complexes, have been the most commonly reported (Table 2). This is due to their emission characteristics showing visible-light luminescence, sharp bands, long emission lifetimes, and large Stokes shifts [82], which could be readily distinguished from background or interfering fluorescence derived from the polymer and other common organic interfering compounds in biological systems. Selected examples of chemosensory applications of the lanthanide-based MIPs are summarized in Table 2. As the emission energy of lanthanides is insensitive to the change of the ligand and micro-environment, the binding events in their MIP chemosensors would only result in quenching or sensitization of their emission (Figure 7a) [83]. Apart from lanthanides, zinc(II) complexes [84,85] and phosphorescent transition metal complexes [79,86] have also been reported but are much less investigated. Unlike the f-f luminescence of lanthanides, the phosphorescence of the transition metal complexes can be readily and systematically modified from the ligand design and is sensitive to the change of the micro-environment. As a result, a shift of the emission color in addition to the turn-on or turn-off of the emission intensity to report the binding event in luminescent chemosensors derived from phosphorescent transition metal complexes has also been developed (Figure 7b) [79]. 

### 4.2. Organic Fluorescent Dyes-Functionalized MIPs

**Figure 8:**
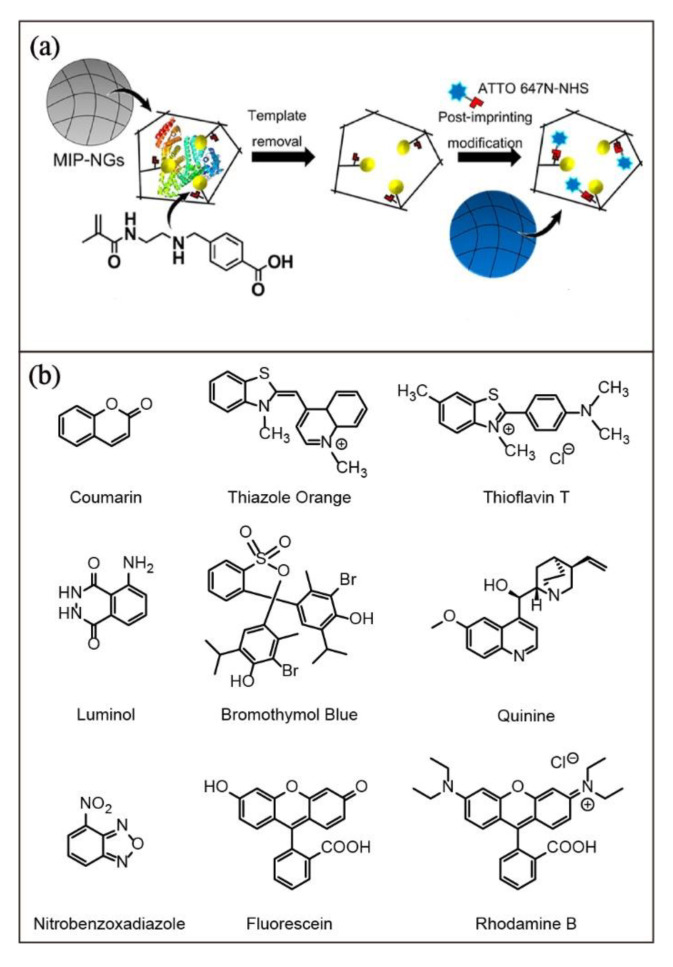
(**a**) Preparation of fluorescent MIP conjugated with ATTO 647N fluorescent dye for porcine serum albumin quantification. Adapted with permission [89]. Copyright 2021, Elsevier. (**b**) Structures of the commonly used organic fluorophores in the development of fluorescent MIPs.

MIPs with organic fluorescent dyes are usually developed by copolymerization of alkene-substituted dyes in the preparation of MIPs, as exemplified in Figure 3a. Apart from copolymerization, the functionalization of MIPs with organic fluorescent dyes has also been reported [88]. Selected examples of organic fluorescent dye-functionalized MIPs are summarized in Table 3. The conjugation of ATTO 647N fluorescent dye in the post-imprinting modification of porcine serum albumin (PAS)-imprinted MIP nanogel is reported by Takeuchi and coworkers [89]. The fluorescent PSA-MIP can serve as a sensor for monitoring and quantifying porcine serum albumin in pork contamination with very high sensitivity, a linear quantification range of 0.25–5 nM, and a limit of detection of 40 pM (Figure 8a). Although fine-tuning the fluorescent properties of each class of fluorescent dyes is challenging, the availability of many different classes of organic fluorescent dyes with different emission characteristics, such as coumarin, thiazole, thioflavin, fluorescein, rhodamine B, bromothymol blue, quinine, luminol and their derivatives (Figure 8b) [57,90,91] have made them popular in designing luminescent MIPs. However, the performance of their chemosensory application may be affected by background fluorescence because the emission lifetimes of these dyes are indistinguishable from the background fluorescence. Moreover, fine-tuning of their emission properties cannot be easily achieved. The poor photostability due to photobleaching of the organic fluorescent dyes is another limitation for prolonged applications [92].

### 4.3. MIPs Encapsulated on Luminescent Nanomaterials

**Figure 9:**
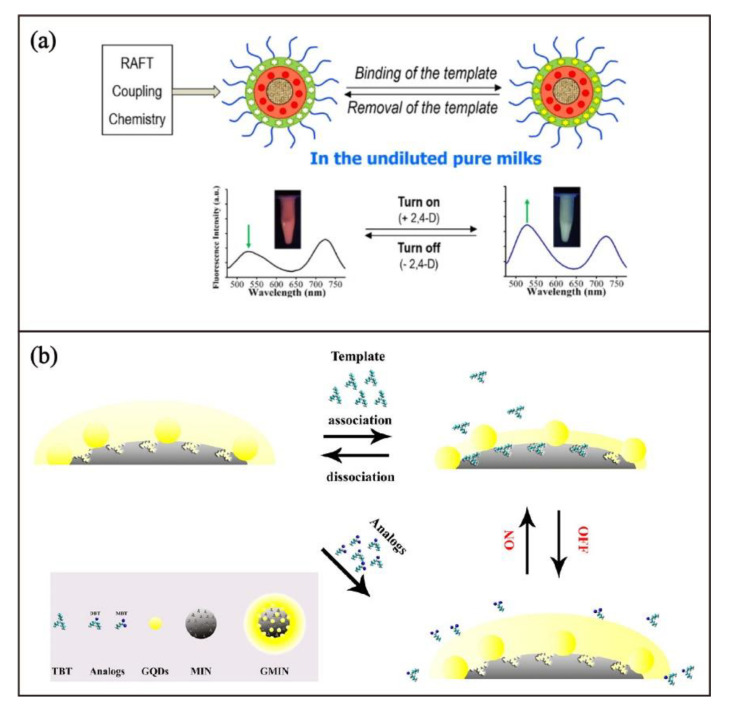
Schematic illustration for (**a**) the preparation of fluorescent MIP-coated CdTe QDs for 2,4-dichlorophenoxy acetic acid detection. Adapted with permission [112]. Copyright 2020, American Chemical Society. (**b**) Preparation and sensing principle of the graphene QDs-immobilized MIP nanocomposite for the detection of tributyltin. Adapted with permission [117]. Copyright 2019, American Chemical Society.

Through encapsulation, as exemplified in Figure 6, luminescent nanomaterials can also be used as binding event reporters for non-luminescent MIPs. The rapid growth of the research works on luminescent nanomaterials in the past decades have led to the development of different types of luminescent nanomaterials, including inorganic semiconductor quantum dots (QDs), noble-metal nanoclusters, organic QDs (carbon dots and graphene QDs) and their doped or coated derivatives [106,107,108]. Inorganic QDs, especially Cd- and Zn-based QDs, are well-recognized as promising nanomaterials due to their three-dimensional quantum confinement, which increases optical nonlinearity [109]. Compared to fluorescent organic dyes, QDs possess more stable and stronger photoluminescent properties with a wide range of excitation and sharp emission bands. Moreover, their emission characteristics can be readily modified by their size [109,110]. Based on QDs-MIP core-shell design, different types of fluorescent MIP chemosensors have been reported [45,46,111,112,113,114,115,116,117,118,119,120,121,122,123,124,125,126,127,128,129,130]. For instance, Zhang and coworkers [112] reported a dual emissive MIP microsphere, composed of a red CdTe QD core and an imprinted MIP shell with the surface-functionalized green fluorescent dye, 4-nitrobenzo-oxadiazole and imprinted cavities for 2,4-dichlorophenoxy acetic acid. After grafting the microparticles with poly(N-isopropylacrylamide) brushes, the MIP can serve as a ratiometric fluorescent sensor for 2,4-dichlorophenoxy acetic acid with a detection limit of 0.13 µM in milk samples because the green fluorescence of 4-nitrobenzo-oxadiazole would be enhanced when the cavities bind with 2,4-dichlorophenoxy acetic acid (Figure 9a).

Due to the concerns about the toxicity of Cd-based QDs, Zn-based QDs, including Mn-ZnS and ZnO QDs, have been developed. Moreover, the luminescence properties and thermal, chemical, and photo-stability of Zn-based QDs can be significantly improved by doping various transition metals. For example, Moreda-Piñeiro and coworkers [113] fabricated a fluorescent MIP sensor based on Mn-ZnS QDs for the detection of cocaine and its metabolites in urine samples to monitor drug abuse. To develop the sensor for cocaine, the Mn-ZnS quantum dots was firstly surface modified by polyethylene glycol and then coated with a MIP by surface imprinting polymerization. With the fluorescence quenching of the Mn-ZnS QDs associated with the binding of the cocaine, it can quantify the cocaine content in clinical samples with a detection limit of 0.076 mg L^−1^. A similar design strategy with surface imprinting polymerization could also apply to luminescent metal nanoclusters such as gold nanoclusters and silver nanoclusters to render “turn off” luminescent chemosensors. This is exemplified in the gold nanocluster-based MIP sensor for bisphenol A [114]. The use of luminescent noble metal nanoclusters may offer advantages, including ultra-small particle sizes <5 nm along with lower cytotoxicity, improved biocompatibility, and photostability.

In recent years, carbon-based QDs, including carbon and graphene QDs, have been explored as superior alternatives to metal-based QDs [115]. Compared with traditional semiconductor QDs, carbon-based QDs have higher water solubility, improved chemical stability, and resistance to photobleaching, as well as readily tunable photoluminescent properties through facile modification [116]. By surface functionalization of MIP nanoparticles, prepared by emulsion polymerization, with graphene QDs, Merkoçi, and coworkers have developed “turn-off” luminescent MIP chemosensors for tributyltin [117]. By depositing graphene QDs-immobilized MIP on the nitrocellulose membrane, a highly sensitive paper-based sensing platform for tributyltin with a detection limit of 0.23 ppt can be developed (Figure 9b). Selected luminescent chemosensors by MIP-hybrid luminescent nanomaterials are summarized in Table 4.

## 5. Applications of Luminescent MIP-Based Chemosensors

As discussed above, a wide variety of luminescent MIP-based sensors developed from different design strategies, luminophores, and sensing mechanisms have been reported. Based on the changes in the emission properties, such as enhancement or quenching of emission intensity, dual emissive signals for ratiometric detection, or shifting of emission color, qualitative and quantitative analysis of the specific target analytes can be achieved. Importantly, chemical sensors for different types of target analytes, from simple metal ions, cations, anions, and small organic molecules to macromolecules, can be developed from these luminescent MIP-based chemosensors. On the other hand, most of these sensors work under different conditions, including aqueous and even biological media. As a result, they have found important applications in the field of environmental monitoring, biotechnology, biomedical sciences, clinical diagnosis, and the food industry, as exemplified below. 

### 5.1. Biosensors

For practical applications, biosensors on solid support materials are fabricated [130]. Different methods, including chemical immobilization of MIP-based sensors on solid support and deposition of MIP-based sensors on solid support by adsorption, have been reported to prepare solid-supported luminescent MIP-based sensors. For solid glass supports, luminescent MIP-based sensors can be fabricated by chemical immobilization on the glass surface by surface polymerization or drop-casting on glass slides [131]. For example, Takeuchi and coworkers used the fluorescent functional monomer dansyl ethylenediamine-conjugated O-acryloyl L-hydroxyproline to synthesize human serum albumin-imprinted polymer on glass substrates by radical polymerization to serve as a protein biosensor [132]. This work represents a promising sensing platform for detecting specific species of protein applied in biotechnology and life sciences.

**Figure 10:**
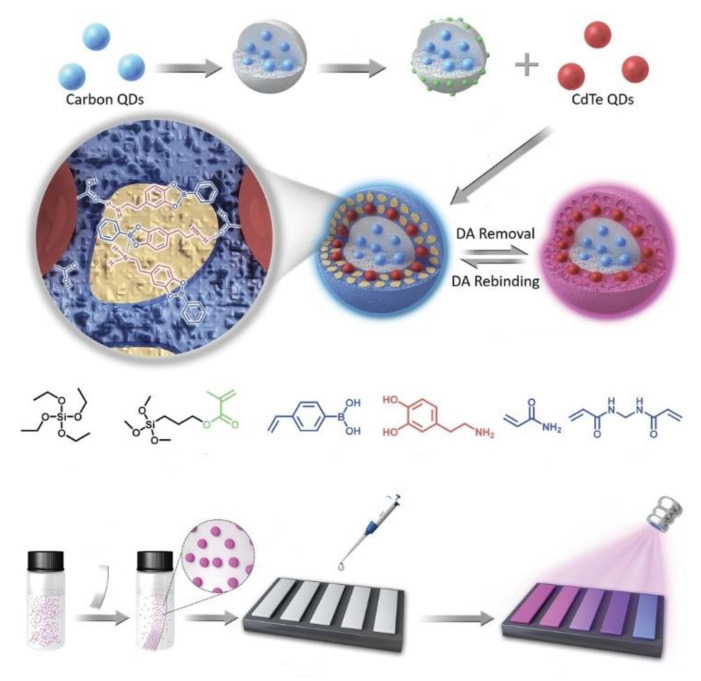
Schematic illustration of molecularly imprinted nanoparticles for dual emission detection of dopamine and the visual readout test strip. Adapted with permission [133]. Copyright 2019, Wiley-VCH.

The visual test strips are commonly made by loading luminescent MIP sensors on filter paper. As shown by Pan and coworkers [133], a dual emissive fluorescent nanoparticle-based MIP dopamine sensor can be loaded on filter paper to give a testing strip for the neurotransmitter dopamine. As the MIP displays a constant blue emission from the carbon QDs and a dopamine-dependent red emission from CdTe QDs, the emission color of the testing strip loaded with the MIPs changes in accordance with the variation of fluorescence of the CdTe QD component upon dopamine binding event. Thus, it can be used for portable visual monitoring of the neurotransmitter dopamine with a detection limit of 100–150 nM. By dropping a biological sample on the test strip, the dopamine levels can be detected within 3 min (Figure 10). 

### 5.2. Bio-Imaging

MIPs have been widely used as antibody mimics due to easy fabrication, well-behaved biocompatibility, high sensitivity, and specificity, as well as little immunogenic response in cells [134]. Luminescent MIPs targeting membrane proteins, including EGFR, HER2, human fibroblast growth factor-inducible 14 (Fn14), P32 receptor, and folate receptor alpha (FRα), representing as disease markers on cell surfaces have been developed [135]. In view of these features, a large number of luminescent MIPs with different biomedical applications are prepared for in vitro and in vivo tracking of the cancer biomarkers and studying the morphologies of the tumor cells [136]. During the therapeutic process, these luminescent MIPs can serve as the antibody substitute to recognize the cancer cells to direct the liberation of drugs [137,138,139].

**Figure 11:**
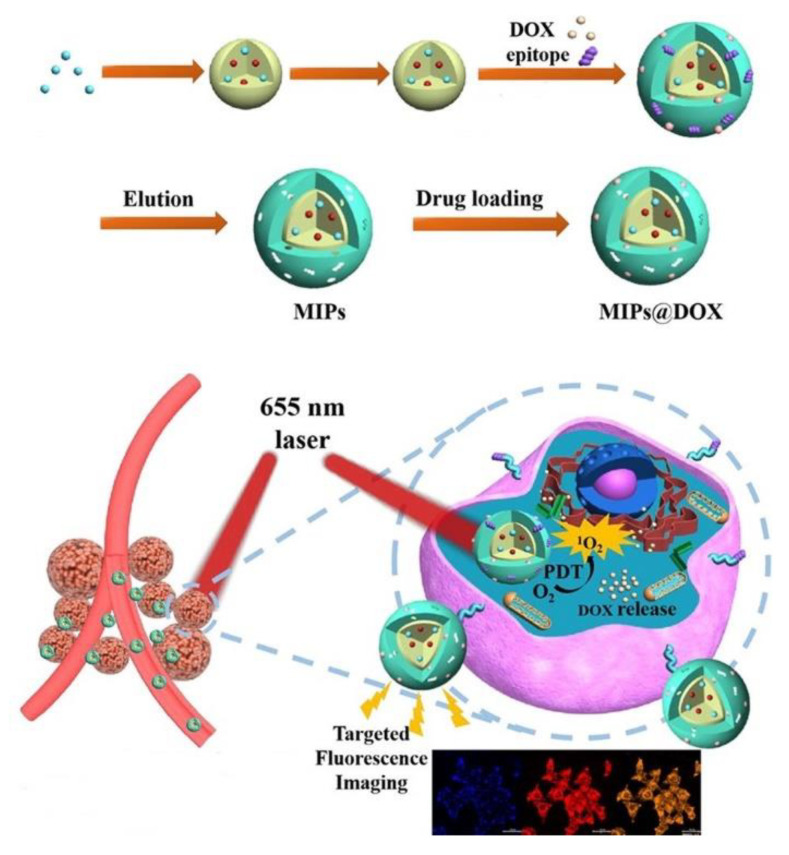
Schematic illustration for the preparation of luminescent MIP@DOX and its targeted chemo-photodynamic synergistic cancer therapy. Adapted with permission [140]. Copyright 2020, American Chemical Society.

For example, Zhang and coworkers have designed and constructed a fluorescent MIP (MIP@DOX) with different functional molecules for simultaneous fluorescence imaging-guided target recognition and chemo-photodynamic therapy [140]. The MIP is designed based on a core-shell structure with the core encapsulating the emissive gadolinium-doped silicon quantum dots (Si_Gd_QDs) and photosensitizer chlorin e6, and surface imprinted polymer with an anticancer drug doxorubicin (DOX) and the epitope peptide of CD59 protein as the templates. With this multifunctional core-shell MIP, the epitope peptides lead to precisely reaching the cancer cells; the emission and magnetic properties of Si_Gd_QDs in the core facilitate the fluorescence and magnetic resonance imaging dual modality; the photosensitizer chlorin e6 can function as a photodynamic therapeutic reagent to generate toxic ^1^O_2_ upon irradiation; and DOX is released to kill the cancer cells (Figure 11).

### 5.3. Food Contaminants and Spoilage Detection

**Figure 12:**
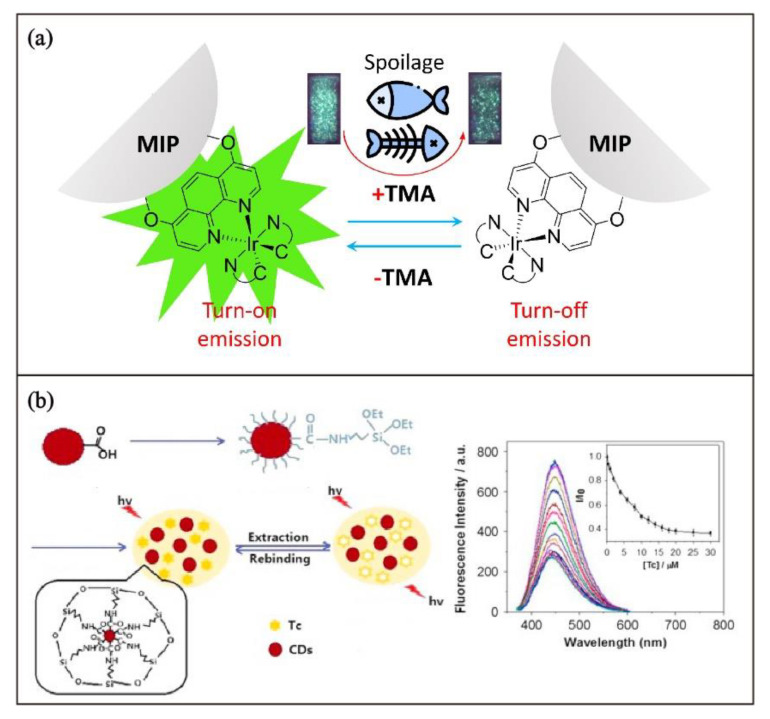
Schematic illustration of (**a**) the luminescent MIP sensor PTMA-Ir for selective detection of TMA released in spoiled seafood. (**b**) Preparation and fluorescence spectral changes of MIP grafted on carbon QDs for selective detection of trace tetracycline in milk. Adapted with permission. [142] Copyright 2016, Elsevier.

The detection of food contaminants and spoilage are important food safety issues. Luminescent MIP-based sensors allow for real-time monitoring of food spoilage. For example, the spoilage of seafood can be monitored by volatile amines, particularly trimethylamine (TMA) vapor. Through surface chemical immobilization of phosphorescent cyclometalated Ir(III) complex on TMA-imprinted polymer (PTMA-Ir), Ko and coworkers have developed luminescent MIP for the quantification of TMA vapor. The surface chemical immobilization strategy was inspired by the method reported by the same research group for the preparation of iridium-based polymer-supported photocatalysts [141]. After the binding of TMA in the imprinted cavities of PTMA-Ir, it results in the quenching of MLCT phosphorescence of the immobilized iridium complexes in close proximity. Since the phosphorescence response of PTMA-Ir toward TMA in the solution state is rapid and sensitive, a testing strip as a visual indicator for seafood spoilage can be made by loading PTMA-Ir on filter paper. As the testing strip shows rapid emission quenching responses within 5 s upon exposure to TMA vapor, it can be used for real-time monitoring of seafood spoilage (Figure 12a). 

Luminescent MIPs have also been developed for the detection of food contaminants. This is exemplified in the luminescent MIP-based tetracycline sensor, which is designed by doping luminescent carbon-QD-containing monomer in the preparation of the tetracycline-imprinted polymer [142]. The luminescent responses of the MIP are highly selective and sensitive toward tetracycline. With a detection limit of 5.45 nM, the MIP can be used for the detection of tetracycline in milk due to the administration of antibiotics in cattle (Figure 12b).

### 5.4. Clinical Diagnosis

**Figure 13:**
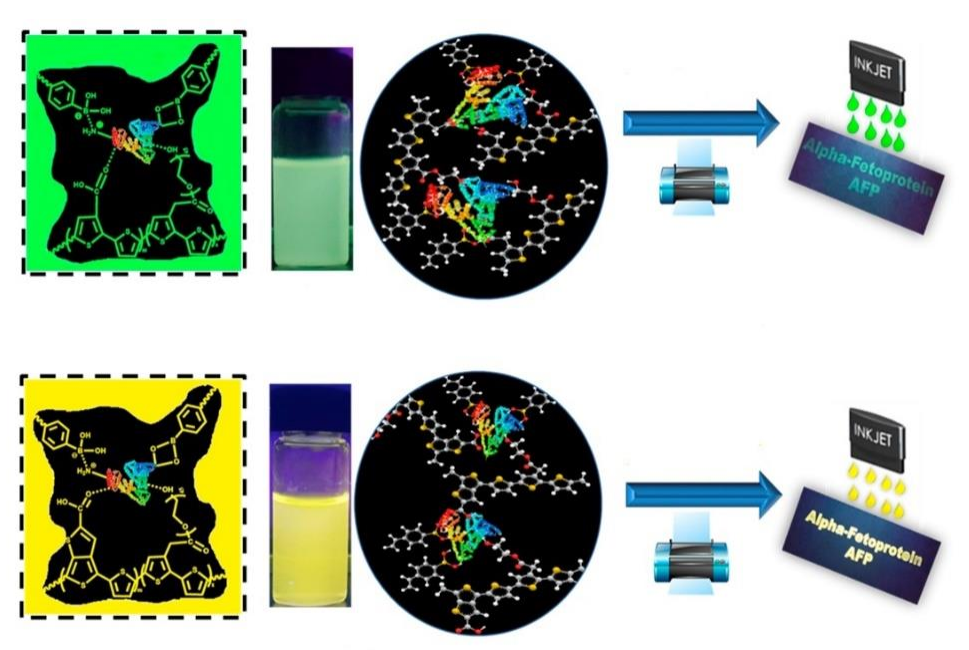
Illustration of the signal readout process of fluorescent conjugated polythiophene-based MIP sensors for α-fetoprotein detection. Adapted with permission [145]. Copyright 2020, Elsevier.

In recent years, the development of luminescent MIPs has provided a promising sensing platform for point-of-care diagnostic applications. The fast, sensitive, high specificity, and simple sampling requirement of luminescent MIP-based sensors have overcome several drawbacks of traditional diagnostic methods, including time-consuming sample preparation and the use of sophisticated instrumentation. Based on the biomarker identified for disease diagnosis, luminescent MIPs for biomarker detection with optical readout signals for simple detection is of great significance for clinical screening [143,144]. For example, Lee and coworkers [145] demonstrated the successful application of an enzyme-free and biocompatible fluorescent MIP-based conjugated polythiophenes for the selective detection of the liver cancer biomarkers, α-fetoprotein, and carcinoembryonic antigen, in blood serum samples, which are hard to detect without complicated sample preparation and precise analytical instrumentation. The emission properties of the polythiophenes can be modified by changing the polythiophene backbone. These MIPs can be printed on filter paper by inkjet printing to produce a point-of-care paper assay kit for cancer detection (Figure 13).

## 6. Conclusions

In this review, the recent design strategies, development, and representative applications of luminescent MIP-based chemosensors over the past ten years have been described and emphasized. Based on the well-developed methodologies, the progress of synthetic MIPs with cavities capable of serving as the synthetic receptors for wide varieties of targeted molecules/ions ranging from metal ions and simple molecules to macromolecules have been discussed. Using various design strategies, different types of luminophores, including organic fluorescent dyes, transition metal complexes, lanthanide complexes, and nanomaterials, can be incorporated onto the surface or backbone of the MIPs to serve as the emissive reporters for the binding events of the cavities in the MIPs. Based on the emissive responses towards the target analyte, these luminescent MIPs can serve as the chemosensors to detect and quantify the analyte. With the robustness and compatibility of the luminescent MIPs to different conditions, they have found promising applications in different areas, including environmental monitoring, biotechnology, biomedical sciences, clinical diagnosis, and the food industry. It is expected that parallel to the growing demands of analytical tools for different analytes. Extensive research works to develop new luminescent MIPs for novel applications will be reported.

Despite outstanding performance and potential applications, the limitations of each strategy in designing luminescent MIPs for chemosensors should be noted for the successful development of luminescent MIP-based sensors with drastic optical responses. In addition to the performance, biocompatibility, and toxicity must be considered when designing MIP-based chemosensors, in particular those aimed at biotechnological and biomedical applications. On the other hand, the long-term chemical inertness of these luminescent MIPs might become an environmental threat when these MIPs are commercialized and widely used. This issue can be tackled by developing biodegradable MIPs, which have received growing attention in recent years. However, biodegradable MIP-based luminescent chemosensors are almost unexplored. 

## Figures and Tables

**Table 1:** Commonly used reagents in the preparation of MIPs.

Components	Examples
Monomer	Acrylic acid
	Methacrylic acid
	2-Vinylpyridine
	Styrene
	4-Vinylaniline
	Methyl methacrylate
	1-Vinylimidazole
	Acrylamide
Crosslinker	Ethylene glycol dimethacrylate
	Divinylbenzene
	1,1,1-Trimethylolpropane trimethacrylate
	1,3-Diisopropenyl benzene
	Pentaerythritol triacrylate
Solvent	Acetonitrile
	2-Methoxyethanol
	Methanol
	Chloroform
	Tetrahydrofuran
	N,N-Dimethylformamide
Initiator	Benzoyl peroxide
	Azobisisobutyronitrile
	Ammonium persulfate
	Ethyl 2-chloro-propionate

**Table 2:** Selected examples of Lanthanide-based luminescent MIP chemosensors.

Lanthanide Complex	Sensing Target	λ_em_	Detection Range	Limit of Detection	Ref.
[Eu(TTA)_3_phen]	Salicylic acid	614 nm	0–724 µM	174 µM	[70]
EuCl_3_	Fluoroquinolone antibiotics	612 nm	0.5–50 µM	0.58 µM	[71]
EuCl_3_·6H_2_O	Picloram herbicide	616 nm	–*^a^*	–*^a^*	[76]
EuCl_3_·6H_2_O	Tenuazonic acid	615 nm	88.4–1040 µM	26 µM	[77]
Tb(NO_3_)_3_·5H_2_O	Melatonin	748 nm	0.004–2.153 nM	0.048 pM	[78]
EuCl_3_·6H_2_O	Pinacolyl methylphosphonate	618 nm	0–4.44 µM	–*^a^*	[80]
[Eu(TTA)_3_phen]	Copper(II)	616 nm	10–100 µM	–*^a^*	[81]
TbCl_3_·6H_2_O	Salicylic acid	545 nm	0.14–72.4 µM	0.290 µM	[87]

*^a^* Not reported.

**Table 3:** Selected examples of organic fluorescent dye-functionalized luminescent MIP chemosensors.

Fluorescent Dye	Sensing Target	λ_em_/nm	Detection Range	Limit of Detection	Ref.
FITC	λ-Cyhalothrin	531	0–60 nM	9.17 nM	[72]
ATTO 647N	Porcine serum albumin	668	0.25–5 nM	40 pM	[89]
FITC	Lysozyme	520	0.1–0.7 µM	–*^a^*	[57]
Nitrobenzoxadiazole	Sialic acid	509	–*^a^*	–*^a^*	[90]
Rhodamine	Hyaluronan	–*^a^*	–*^a^*	–*^a^*	[93]
Luminol	Chrysoidine	–*^a^*	0.1–10 µM	0.032 µM	[94]
Coumarin	Tamoxifen	521	–*^a^*	10 mM	[95]
ATTO 647N	Human serum albumin	664	12–192 nM	13 nM	[96]
Coumarin	4-Nitrophenol	461	0.001–7.5 µM	0.5 nM	[97]
Thioflavin T	Guanosine	488	–*^a^*	5 µM	[98]
Nitrobenzoxadiazole	2,4-Dichlorophenoxyacetic acid	528	0.56–80 µM	90 nM	[99]
Nitrobenzoxadiazole	Phospholipids	512	18–60 µM	5.6 µM	[100]
FITC	Trypsin	515	–*^a^*	50 pM	[101]
FITC	17β-Estradiol	–*^a^*	0.10–70 µM	0.03 uM	[102]
FITC	Doxycycline	520	0.2–6 µM	117 nM	[103]
Methyl red	Dimethoate	450	0.6–34 nM	18.3 pM	[104]
Rhodamine	4-Nitrophenol	582	0.01–10 µM	3.0 nM	[105]

*^a^* Not reported.

**Table 4:** Selected examples of typical luminescent nanomaterials-based MIP chemical sensors.

Luminescent Nanomaterials	Sensing Target	λ_em_/nm	Detection Range	Limit of Detection	Ref.
CdTe QDs	2,4-Dichlorophenoxy acetic acid	528	0–15 μM	0.28 μM	[112]
Mn-ZnS QDs	Cocaine	590	0–3.296 μM	0.250 μM	[113]
AuNCs	Bisphenol A	–*^a^*	0–13.1 μM	0.10 μM	[114]
Carbon QDs	Promethazine hydrochloride	431	2.0–250 μM	0.5 μM	[116]
Graphene QDs	Tributyltin	440	0.687 pM–0.687 nM	0.79 pM	[117]
ZnO QDs	Dimethoate	536	0.087–13.92 μM	0.026 μM	[118]
AuNCs	Erythromycin	585	0.1 μM–11.9 μM	12 nM	[119]
Carbon QDs	Ofloxacin	614	1–50 nM	0.25 nM	[120]
Nitrogen CDs	2,4,6-Trinitrophenol	408	0.5–2.5 nM	0.15 nM	[121]
CdTe QDs	Tetracycline	–*^a^*	0.5–15 µM	0.14 µM	[122]
ZnO QDs	Methylene blue	554	0–100 µM	1.27 µM	[123]
CsPbBr_3_ QDs	Omethoate	510	0.23–1.88 pM	0.09 pM	[124]
Graphene QDs	Methamphetamine	420	5–50 µM	0.011 µM	[125]
Carbon QDs	N-Acyl homoserine lactones	–*^a^*	2.66–127 nM	0.033 nM	[126]
Carbon QDs	Tannic acid	440	1–200 nM	0.6 nM	[127]
Mn-ZnS QDs	Bilirubin	590	10.99–63.84 µM	1.8 µM	[128]
Cu-Mn-ZnS QDs	Folic acid	490, 595	0.01–5 µM	6 nM	[129]

*^a^* Not reported.

## Data Availability

No new data were created or analyzed in this study.
